# Optimization of Production Conditions for Protoplasts and Polyethylene Glycol-Mediated Transformation of *Gaeumannomyces tritici*

**DOI:** 10.3390/molecules23061253

**Published:** 2018-05-24

**Authors:** Mei Wang, Jie Zhang, Lanying Wang, Lirong Han, Xing Zhang, Juntao Feng

**Affiliations:** 1 Research and Development Center of Biorational Pesticide, Northwest A&F University, Yangling 712100, China; mmeiwang@126.com (M.W.); deit1984@sina.com (J.Z.); daivemuwly@126.com (L.W.); hlr4119@126.com (L.H.); 2 Institute of Tropical Agriculture and Forestry, Hainan University, Haikou 570228, China; 3 Engineering and Research Center of Biological Pesticide of Shaanxi Province, Yangling 712100, China

**Keywords:** *Gaeumannomyces tritici*, protoplast, PEG-mediated, transformation, response surface methodology (RSM)

## Abstract

Take-all, caused by *Gaeumannomyces tritici*, is one of the most important wheat root diseases worldwide, as it results in serious yield losses. In this study, *G. tritici* was transformed to express the hygromycin B phosphotransferase using a combined protoplast and polyethylene glycol (PEG)-mediated transformation technique. Based on a series of single-factor experimental results, three major factors—temperature, enzyme lysis time, and concentration of the lysing enzyme—were selected as the independent variables, which were optimized using the response surface methodology. A higher protoplast yield of 9.83 × 10^7^ protoplasts/mL was observed, and the protoplast vitality was also high, reaching 96.27% after optimization. Protoplasts were isolated under the optimal conditions, with the highest transformation frequency (46–54 transformants/μg DNA). Polymerase chain reaction and Southern blotting detection indicated that the genes of hygromycin phosphotransferase were successfully inserted into the genome of *G. tritici*. An optimised PEG-mediated protoplast transformation system for *G. tritici* was established. The techniques and procedures described will lay the foundation for establishing a good mutation library of *G. tritici* and could be used to transform other fungi.

## 1. Introduction

*Gaeumannomyces tritici*, one of the major soil-borne pathogens, survives saprophytically on crop debris. *G. tritici* colonizes roots of susceptible cereals, especially wheat, and causes take-all disease in the affected areas [1]. The typical symptom is black necrosis on roots after infection by *G. tritici* mycelium [2,3]. This devastating disease is widespread throughout wheat producing areas worldwide [4,5], resulting in yield losses of 40–60% [6,7,8]. However, chemical fungicides are not always effective for soil-born fungi, and theses fungicides may have negative effects on the environment and human health [9,10]. The *G. tritici* genome sequence is available [1] and there is an urgent need for an efficient and rapid transformation system.

The polyethylene glycol (PEG)-mediated protoplast transformation technique is the most common approach for transformation of filamentous fungi [11,12,13]. This technique has advanced our ability to investigate this intractable fungus at the molecular level. The major limiting factor for the PEG-mediated transformation protocol for *G. tritici* is the production of protoplasts. Transformation of protoplasts has considerable potential for strain improvement and genetic analysis, providing an opportunity to rapidly study a large number of genes. Establishment of protoplast transformation techniques for *G. tritici* is of crucial importance to biotechnology. Stanway and Buck reported the viral infection of *G. tritici* protoplasts [14]. Henson and Park used polyethylene glycol (PEG)-CaC1_2_ mediated transformation of protoplasts for *G. tritici* [15,16]. Bowyer reported the mutagenesis of protoplasts in *G. tritici* by 4-nitroquinolene oxide [17]. Although protocols for transformation of *G. tritici* DNA have been reported, we obtained transformants with a low transformation frequency when we used these protocols. Consequently, we developed a new and efficient method for the transformation of *G. tritici*, and the parameters involved in production of protoplasts were optimized.

Optimization of conditions for protoplast production using the classical method involves changing one independent variable while the other factors remain constant. The conventional methods for multifactor experimental design are time-consuming and inefficient for detecting the true optimum, especially given the interactions between the factors [18,19]. The response surface methodology (RSM) is well-suited for optimizing and studying the interactions among distinct factors using a minimum number of experiments [20,21]. RSM includes factorial design and regression analysis that can be used to construct models to determine interactions, explore optimum conditions of factors under study for desirable responses, and assess the relationship between a set of controllable experimental factors and observed results [22,23].

The objective of the present research was to optimize the conditions to maximise the yield of protoplasts using both one-factor-at-a-time optimization and the response surface methodology. The optimal values of these factors were obtained using response surface methodology. Moreover, the hygromycin phosphotransferase gene (*hph*) fragment of plasmid pSilent-1 was transformed into the genomic DNA of *G. tritici* by PEG-mediated transformation of protoplasts, which laid the foundation for establishing a good mutation library of *G. tritici*.

## 2. Results

### 2.1. Effects of Enzyme on Protoplast Yield

The results showed that a mixture of 0.25% snailase (S), 0.25% driselase (D), and 0.25% lysing enzyme (L) produced 2.68 × 10^7^ protoplasts/mL, which was higher than the enzyme of 0.25% L (2.07 × 10^7^ protoplasts/mL). The enzyme of 0.25% L was significantly higher than the enzyme of 0.25% D (0.17 × 10^7^ protoplasts/mL) and 0.25% S (0.03 × 10^7^ protoplasts/mL). There was no significant difference between the enzyme of 0.25% L (2.07 × 10^7^ protoplasts/mL), 0.25% S and 0.25% L (2.10 × 10^7^ protoplasts/mL), and 0.25% L and 0.25% D (2.37 × 10^7^ protoplasts/mL) (Table 1).

### 2.2. Effects of Media on Protoplast Yield

**Figure 1:**
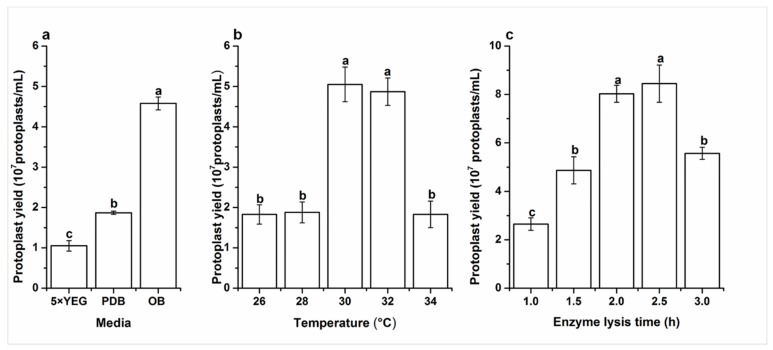
(**a**) Effects of media on protoplast yield; (**b**) effects of digesting temperature on protoplast yield; and (**c**) effects of enzyme lysis time on protoplast yield. Data represent the mean ± standard errors (SE) of three independent experiments. Different lower-case letters indicate a significant difference (*p* < 0.05).

The yield of the protoplasts of the mycelia of *G. tritici* inoculated into media of 5× YEG, PDB, and OB were 1.05 × 10^7^, 1.87 × 10^7^, and 4.58 × 10^7^ protoplasts/mL, respectively (Figure 1a). The results showed that OB was the best medium for preparing the protoplasts of *G. tritici*.

### 2.3. Effects of Digesting Temperature on Protoplast Yield

The results showed that temperatures from 30 to 32 °C were the best for enzyme activity to obtain protoplasts. When the temperature was below 30 or above 32 °C, the activity of the lysing enzyme greatly decreased, which significantly affected the protoplast yield (Figure 1b).

### 2.4. Effects of Enzyme Lysis Time on Protoplast Yield

The mycelia of *G. tritici* were digested by 0.5% lysing enzyme at 30 °C. Figure 1c shows that the protoplast yield significant increased with the increase in digesting time. However, the number of protoplasts decreased rapidly after an enzymatic digesting time of 2.5 h. There was no significant difference in protoplast production between the digesting time of 2.0 (8.03 × 10^7^ protoplasts/mL) and 2.5 h (8.45 × 10^7^ protoplasts/mL). Therefore, the best digesting time for the 0.5% lysing enzyme is between 2.0 and 2.5 h.

### 2.5. Effects of Mycelia Weight on Protoplast Yield

**Figure 2:**
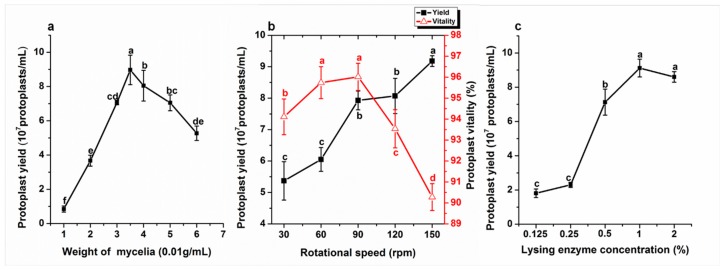
(**a**) Effects of mycelia weight on protoplast yield; (**b**) effects of rotational speed on protoplast isolation; and (**c**) effects of concentration of lysing enzyme on protoplast yield. Data represent the mean ± standard errors (SE) of three independent experiments. Different lower-case letters indicate significant difference (*p* < 0.05).

The mycelia of *G. tritici* were digested by 0.5% lysing enzyme at 30 °C for 2 h. The yield of protoplasts reached a maximum when 1 mL 0.5% lysing enzyme was reacted with 0.035 g mycelia (Figure 2a). The protoplast yield significantly decreased when the 1 mL 0.5% lysing enzyme contained less than or higher than 0.035 g mycelium. Therefore, the optimal weight of mycelia was 0.035 g in 1 mL 0.5% lysing enzyme for producing protoplasts.

### 2.6. Effects of Rotational Speed on Protoplast Isolation

The protoplast yield from mycelia incubated on a rotary shaker at 30, 60, 90, 120, and 150 rpm at 30 °C for 2 h were 5.37, 6.05, 7.93, 8.07, and 9.18 × 10^7^ protoplasts/mL, respectively. The results showed that when the rotational speed was 150 rpm, the yield of protoplasts was significantly higher than for other rotational speeds. There was no significant difference between the rotational speeds of 90 and 120 rpm. Although the yield of protoplasts at 150 rpm was higher than at other rotational speeds, the vitality was 90.28%. When the rotational speed was 90 rpm, the protoplast vitality was highest, reaching over 96%, and the yield was also higher, reaching 7.93 × 10^7^ protoplasts/mL. When the rotational speed was 150 rpm, the yield of protoplast was the highest, but protoplast viability decreased significantly compared with 90 rpm. In summary, considering protoplast yield and protoplast viability, the best rotational speed was 90 rpm (Figure 2b).

### 2.7. Effects of Concentration of Lysing Enzyme on Protoplast Yield

The results presented in Figure 2c reveal that 0.5% lysing enzyme produced 7.13 × 10^7^ protoplasts/mL, which was significantly higher than the 0.125% lysing enzyme (1.81 × 10^7^ protoplasts/mL) and 0.25% lysing enzyme (2.30 × 10^7^ protoplasts/mL). The protoplast yields with different concentrations of lysing enzyme (1% and 2%) were 9.12 × 10^7^ and 8.60 × 10^7^ protoplasts/mL, respectively. There was no significant difference between 1 and 2% lysing enzymes for producing protoplasts (Figure 2c).

### 2.8. Optimization of Production Conditions for Protoplasts by RSM

The Box–Behnken design was used to determine the optimal conditions of protoplast production in *G. tritici*. The levels of the three independent variables, temperature, enzyme lysis time, and concentration of lysing enzyme, are provided in Section 4.5. A total of 15 experiments with different combinations of three independent variables were performed according to the Box–Behnken design (Table 2). The protoplast yields of the experimental and predicted values are provided in Table 2, and the predicted values were calculated from the quadratic polynomial equation mentioned below, which was used to explain the relationship between the protoplast yield and independent variables

Y= 9.37 + 1.26X_1_ – 0.12X_2_ + 0.15X_3_ − 0.52X_1_X_2_ + 0.18X_1_X_3_ − 0.10X_2_X_3_ − 1.53X_1_^2^ − 0.33X_2_^2^ − 0.84X_3_^2^ (1)

where Y is the predicted response of protoplast yield; and χ_1_, χ_2_, and χ_3_ are the coded values of temperature, enzyme lysis time, and lysing enzyme concentration, respectively.

The analysis of variance (ANOVA) of the quadratic regression model demonstrated that the model was significant due to the Fisher’s *F* test (*F*_model_ = mean square regression/mean square residual = 29.06) with a very low *p* value, with (*P*_model_ > *F*) = 0.0009 (Table 3). The fit of the model was checked using the determination coefficient, *R*^2^, which was 0.9812, indicating that 98% of the variability in the response could be explained by the model. The coefficient of variation (CV) was related to the reliability and precision, and the higher the value of CV, the lower the reliability of the experiment [24]. In this model, a lower CV value (3.89%) indicated better reliability and precision of the experiment. The results showed that the linear coefficient χ_1_ was more significant than the other factors, which implied that the temperature strongly influenced protoplast yield. The interaction coefficients of χ_1_χ_2_, χ_1_χ_3_, and χ_2_χ_3_ seemed to be insignificant, which revealed that the interactions of any two variables insignificantly affected protoplast yield.

**Figure 3:**
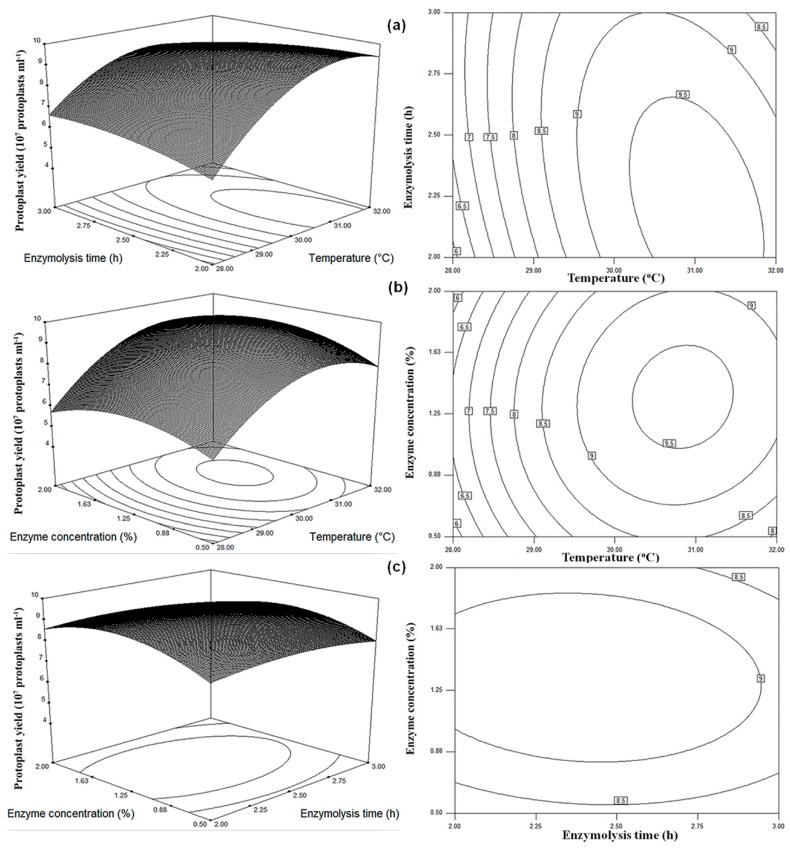
Response surfaces and contour plots showing: (**a**) the combined effect of temperature and enzyme lysis time on the protoplast yield; (**b**) the combined effect of temperature and lysing enzyme concentration on the protoplast yield; and (**c**) the combined effect of enzyme lysis time and lysing enzyme concentration on the protoplast yield.

The three-dimensional (3D) response surface plots and the two-dimensional (2D) contour plots described by the regression model were designed to expose the optimal values of the independent variables and the interactive effects of each independent variable on the response [24]. From the 3D response surface plots and the corresponding 2D contour plots, the optimal values of the independent variables and the maximum responses were predicted and the interactions between each independent variable were explained (Figure 3). Each contour curve represented a response value estimated by the pairwise combination with another factor maintained at its zero level. From Figure 3, the optimal values of the conditions for producing the maximum yield of protoplasts lay in the following ranges: temperature 30–32 °C (Figure 3a,b), enzyme lysis time 2–2.5 h (Figure 3a,c), and lysing enzyme concentration 0.88–1.63% (Figure 3b,c). By performing analyses with Design Expert Software, the optimal values of the independent variables in actual units were: temperature 31 °C, enzyme lysis time 2.2 h, and lysing enzyme concentration 1.4%. The model predicted that the maximum protoplast yield of 9.76 × 10^7^ protoplasts/mL would be obtained by using the above optimal conditions.

A verification experiment was performed using the optimized conditions. The experiment demonstrated that the value of the experimental response (9.83 × 10^7^ protoplasts/mL) was almost equal to the maximum predicted yield (9.76 × 10^7^ protoplasts/mL), clearly proving the aptness of model under these conditions. Consequently, the model was considered to be accurate and reliable for predicting the protoplast yield. Moreover, the protoplast vitality was high, reaching 96.27% under the optimized conditions.

### 2.9. Transformation

To regenerate the transformed protoplasts, they were initially plated onto potato dextrose agar (PDA) plates with 120 μg/mL hygromycin B using toothpicks, and continuously transferred five times onto new PDA plates containing 120 μg/mL hygromycin B. More than 98% transformants were able to grow stably, indicating that we had obtained positive transformants. There were 46–54 transformants/μg NDA (Table 4).

### 2.10. Southern Blotting

**Figure 4:**
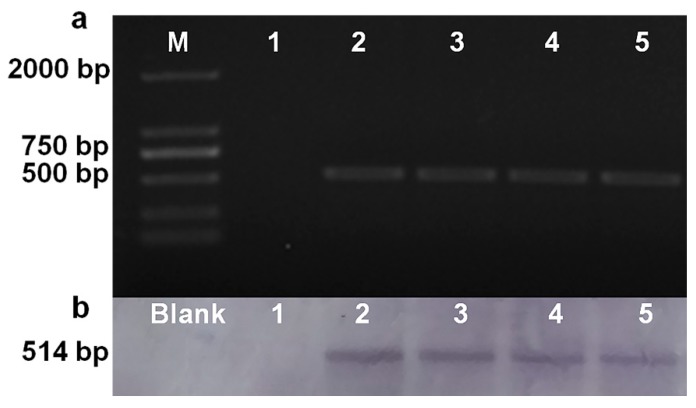
Southern blot hybridization analysis of strains using the region of hygromycin phosphotransferase gene as a probe. (**a**) Strains were identified by polymerase chain reaction (PCR) with primers of Yzb-F/R using genomic DNA as the template; (**b**) Southern blot hybridization analysis of strains using the region of hygromycin phosphotransferase gene as a probe, which was amplified with primers of Yzb-F/R. Lane M: DNA Marker 2000; Lane 1: *G. tritici*; Lanes 2–5: *hph* transformants.

The integration of *hph* into the genome of transformants was verified using PCR and Southern blotting. As showed in Figure 4a, diagnostic PCR with YzbF/R primers, using genomic DNA of hygromycin-resistance transformants as the template, showed that a 514-bp product was amplified, whereas no product was found using untransformed *G. tritici* genomic DNA. Southern blot analyses were evaluated with the probe. The probe detected a single band with an expected size of 514 bp in the four transformants, and no band was found in the wild type (Figure 4b). The results indicated that the DNA fragment (1790 bp) of the hygromycin-resistant gene was transformed into the genome of the transformants.

## 3. Discussion

Since the first report of a DNA-mediated transformation for a fungus in 1973 [25], many fungal species, such as *Sclerotinia sclerotiorum* and *Aspergillus oryzae*, have been transformed not only for PEG-mediated transformation of protoplast, but also for *Agrobacterium*-mediated, biolistics, and electroporation transformation [26,27,28]. PEG-mediated protoplast transformation is an important method for studying gene function in filamentous fungi [13]. The preparation of the protoplasts is a pivotal step in PEG-mediated protoplast transformation for filamentous fungi, and the quality and quantity of protoplasts are very important for successful transformation [26]. Many factors and conditions during protoplast preparation affect the quantity and quality of the protoplasts obtained. Notably, the conditions of protoplast formation for different fungi are not identical according to the individual characteristics, such as genetic background, physiological and biochemical characteristics, and cell wall structure. In this study, to obtain a large number of high quality protoplasts of *G. tritici*, a series of experiments were systematically performed to determine the optimum conditions for preparing protoplasts of *G. tritici*.

Our results showed that the protoplast generation was poor from PDB or 5× YEG fungal culture medium. However, using the OB medium could improve the generation of protoplasts. Lysing enzymes play an important role in protoplast generation. At present, researchers can select from a diverse variety of commercially available enzymes. Our study also revealed that lysing enzyme had a better effect compared with driselase and snailase, and no significant difference was observed between the enzyme of 0.25% L, the mix of 0.25% S and 0.25% L, and 0.25% L and 0.25% D. The mix of 0.25% S 0.25% D, and 0.25% L produced 2.68 × 10^7^ protoplasts/mL, which was higher than the enzyme of 0.25% L (2.07 × 10^7^ protoplasts/mL) (Table 1). Taking into account economic efficiency, we recommend using lysing enzyme in protoplast generation, and the best digesting time was between 2.0 and 2.5 h. After one-factor-at-a-time analysis, the initial optimum conditions were as follows: 1 mL of 1–2% lysing enzyme reacting with 0.035 g of hyphae at 30–32 °C for 2.0–2.5 h at 90 rpm. The optimal conditions of temperature, enzyme lysis time, and lysing enzyme concentration were further optimized by RSM based on the Box–Behnken design. RSM proved to be an effective statistical tool for optimizing protoplast yield in *G. tritici*. The RSM model equation revealed that temperature, enzyme lysis time, and lysing enzyme concentration were positively significant factors for protoplast production. From the equation, the interactions between two factors were also determined. A positive interaction was calculated between temperature and lysing enzyme concentration, whereas both temperature and lysing enzyme concentration interacted negatively with enzyme lysis time. The final optimized conditions were determined to be: temperature 31 °C, enzyme lysis time 2.2 h, and lysing enzyme concentration 1.4%. Theoretically, the predicted value of protoplast production could reach 9.76 × 10^7^ protoplasts/mL under these conditions. In practice, the maximum protoplast yield was found to be 9.83 × 10^7^ protoplasts/mL in the verification test, which proved that the model was accurately able to predict protoplast yield.

In this study, we demonstrated the expression of the *hph* gene from plasmid pSilent-1 using the PEG-mediated protoplast transformation system described above. The present study examined the susceptibility of protoplasts to hygromycin B. The results showed that *G. tritici* was sensitive to high concentrations of hygromycin B (120 µg/mL for 100% death; Table S1). Thus, hygromycin B was used as a selection system to develop a PEG-based transformation system for *G. tritici*.

We demonstrated that the concentration of protoplast in the transformation process should be appropriate, and that the efficiency of transformation was very low when protoplast concentration was lower than 10^6^ protoplasts/mL or higher than 10^8^ protoplasts/mL. The most suitable protoplast concentration for filamentous fungal transformation was 10^7^ protoplasts/mL [29]. PEG-mediated transformation of the protoplast system increases the binding of cells and DNA, being more sensitive and easier to obtain positive transformants. PEG-mediated transformation provides insights into the usefulness of the system for future studies of gene expression in fungal cells [30]. Comparisons between species are hampered by the fact that different fragment lengths are used for transformation experiments [31,32]. We described the parameters for transformation of *G. tritici* that resulted in a high number of transformants and up to 46–54 transformants/μg DNA. However, *S. sclerotiorum* produced 60–85 transformants/μg DNA [26], and the efficiency of transformation was 40–50 transformants/μg DNA in *Fusarium graminearum* [29].

## 4. Materials and Methods

### 4.1. Fungal Strain, Media, and Reagents

*Gaeumannomyces tritici* (number ACCC30310) was provided by the Agricultural Culture Collection of China (ACCC, Beijing, China). The fungus was grown on potato dextrose agar (PDA) at 25 °C for 5–7 days before being used. The strain was stored at −80 °C by cryopreservation in 30% glycerol solution [33].

The following media were used in this study: PDA (200 g potato, 17 g agar, 20 g dextrose, and 1000 mL distilled water), potato dextrose broth (PDB) (200 g potato, 20 g dextrose, and 1000 mL distilled water), oatmeal broth (OB) (50 g oatmeal and 1000 mL distilled water), 5× yeast extract glucose (5× YEG) (5 g yeast extract, 5 g tryptone, 10 g glucose, and 1000 mL distilled water), CM (10 g dextrose, 2 g peptone, 1 g yeast extract, 1 g acid hydrolyzed casein, 6 g NaNO_3_, 0.5 g KCl, 1 g MgSO_4_·7H_2_O, 1.5 g KH_2_PO_4_, and 1000 mL distilled water), STC (200 g sucrose, 100 mL 0.5 M Tris-Cl pH 8.0, 5.55 g CaCl_2_, and 1000 mL distilled water), 40% PTC (40 g PEG8000 and 100 mL STC), TB_3_ (3 g yeast extract, 3 g acid hydrolyzed casein, 200 g sucrose, and 1000 mL distilled water), bottom agar (3 g yeast extract, 3 g acid hydrolyzed casein, 200 g sucrose, 10 g agar, and 1000 mL distilled water), and top agar (3 g yeast extract, 3 g acid hydrolyzed casein, 200 g sucrose, 15 g agar, and 1000 mL distilled water). All the above media were autoclaved at 120 °C for 20 min. Mycelia lysate (0.5%) was sterilized by filtration (200 mg lysing enzyme and 40 mL 1 mol/L sorbitol).

Hygromycin B was purchased from Sangon Biotech (Shanghai, China). Lysing enzyme (L), driselase (D), and fluorescein diacetate were purchased from Sigma-Aldrich (Guangzhou, China). Snailase (S) and ampicillin (amp) were purchased from Solarbio (Beijing, China).

### 4.2. Hygromycin Phosphotransferase Gene (hph) Fragment

A 1790 bp *PtrpC-hph* fragment containing *hph* under the control of *trpC* gene promoter was amplified from plasmid pSilent-1 [34,35,36]. The *hph* fragment was polymerase chain reaction (PCR)-amplified from pSilent-1 using the primers HygbF (5′-TAACCGTATTACCGCCTTTG-3′) and HygbR (5′-TCGGCATCTACTCTATTCCTTT-3′) in a total reaction volume of 25 μL containing 0.75 μL PCR forward primer (10 μM), 0.75 μL PCR reverse primer (10 μM), 1 μL DNA sample of plasmid pSilent-1, 10 μL RNase free dH_2_O, and 12.5 μL PrimeSTAR Max (Takara, Dalian, China). The reaction mixtures were amplified for 32 cycles (98 °C for 10 s, 55 °C for 5 s, and 72 °C for 2 min) in a C1000TM thermal cycler (BIO-RAD, Shanghai, China). The PCR products were purified by TIANquick Midi Purification kit (Tiangen, Beijing, China).

### 4.3. Preparation of Protoplasts

Mycelia of *G. tritici* were harvested from a seven-day-old PDA culture and inoculated into 50 mL of OB and incubated on a rotary shaker (175 rpm) at 25 °C for 2 days. Then 50 mL CM was added and incubation continued on a rotary shaker (175 rpm) at 25 °C for 2 days. The mycelia were filtered and washed twice with 1 mol/L sorbitol, and mixed with mycelia lysate (40 mL), then incubated on a rotary shaker (90 rpm) at 30 °C for 2 h. Protoplasts were filtered with a layer of cloth and two layers of lens cleaning tissue (Hangzhou Special Paper Industry Co., Ltd., Hangzhou, Zhejiang, China), and centrifuged for 5 min at 3000× *g*. The supernatant was removed, the protoplasts were suspended with 20 mL STC, and centrifuged for 5 min at 3000× *g*. The protoplasts were resuspended with 2 mL STC and stored on ice until transformation.

### 4.4. Protoplast Yield Optimization

#### 4.4.1. Effects of Enzyme on Protoplast Yield

Various enzymes and enzyme combinations were used, including 0.25% lysing enzyme (L), 0.25% driselase (D), 0.25% snailase (S), 0.25% L+ 0.25% D, 0.25% L+ 0.25% S, 0.25% D+ 0.25% S, and 0.25% L+ 0.25% D+ 0.25% S. The other operations were the same as in Section 4.3.

#### 4.4.2. Effects of Media on Protoplast Yield

Mycelia of *G. tritici* were harvested from a seven-day-old PDA culture and inoculated into 50 mL of PDB, OB, and 5× YEG, and incubated on a rotary shaker (175 rpm) at 25 °C for two days. Then, 50 mL CM was added and incubation continued on a rotary shaker (175 rpm) at 25 °C for two days, and the other operations were the same as outlined in Section 4.3.

#### 4.4.3. Effects of Digesting Temperature on Protoplast Yield

Mycelia of *G. tritici* were tested at digesting temperatures of 26, 28, 30, 32, and 34 °C. The other operations were the same as in Section 4.3.

#### 4.4.4. Effects of Enzyme Lysis Time on Protoplast Yield

The mycelia of *G. tritici* were digested for 1, 1.5, 2, 2.5, and 3 h. The other operations were the same as in Section 4.3.

#### 4.4.5. Effects of Mycelia Weight on Protoplast Yield

*G. tritici* mycelial weights of 0.01, 0.02, 0.03, 0.035, 0.04, 0.05, and 0.06 g were digested in 1 mL 0.5% lysing enzyme. The other operations were the same as in Section 4.3.

#### 4.4.6. Effects of Rotational Speed on Protoplast Isolation

On a rotary shaker, mycelia of *G. tritici* were digested at rotational speeds of 30, 60, 90, 120, and 150 rpm. The other operations were the same as in Section 4.3. Protoplasts viability was simultaneously assessed according to the method described by Jia et al. [37].

#### 4.4.7. Effects of Concentration of Lysing Enzyme on Protoplast Yield

Use of a proper enzyme system is usually a crucial for preparing highly efficient protoplasts. A series of concentrations of lysing enzyme (0.125%, 0.25%, 0.5%, 1%, and 2%) were tested. The other operations were the same as in Section 4.3.

### 4.5. Response Surface Optimization Designs

The main purpose of the Box–Behnken design of the experiment was to investigate the relationship between the multiple process variables and protoplast yield in order to determine the optimum conditions for the protoplast production. According to a series of single factor experimental results, three major factors—temperature (X_1_), enzyme lysis time (X_2_), and concentration of lysing enzyme (X_3_)—were selected as the independent variables. Table 5 shows the three experiment levels of the variables. The software Design Expert version 8.05 including a built-in analysis of variance was employed for the experimental design, data analysis, and modeling. A total of 15 experiments were performed, including three center points (Table 2).

### 4.6. Transformation

To 200 µL of protoplast in 50 mL tubes were added 4, 6, 8 µg DNA, respectively, mixed gently and incubated at room temperature for 20 min. Then 1.25 mL 40% PTC (filtration sterilization) was added, mixed gently, and incubated at room temperature for 20 min. Added 10 mL of TB_3_ (50 µg/mL of amp), incubated on a rotary shaker (100 rpm) at 25 °C for 12 h, and centrifuged for 5 min at 3000× *g*. Discarded supernatant and suspended 200 µL of STC. Then added 10 mL bottom agar (60 µg/mL hygromycin B), mixed gently and poured into a Petri dish. Cultured 12 h at 25 °C and added 10 mL top agar (120 µg/mL hygromycin B) to the Petri dish. After 4–5 days, the plates were screened for transformants, which were transferred to PDA media.

### 4.7. Southern Blotting

The wild strain and four transformants of *G. tritici* were grown on petri dishes containing PDA at 25 °C for seven days before they were harvested in sterile distilled water. The mycelia were then transferred to a triangular flask containing 100 mL PDB and incubated on a rotary shaker (175 rpm) at 25 °C for five days. The mycelia were collected by vacuum filtration, and genomic DNA was extracted by Plant Genomic DNA Kit (CWBIO, Beijing, China). The primers YzbF (5′-GATCGTTATGTTTATCGGCACT-3′) and YzbR (5′-TGGCGACCTCGTATTGG-3′) were used for testing and verifying PCR amplification of the *hph* gene fragment.

Southern blot analyses of transformants were performed with a DIG-High Prime DNA Labeling and Detection Starter Kit I (Roche, Shanghai, China) [38]. For Southern blotting of transformants, 15 μg DNA from the wild type and four transformants were digested overnight with HindIII (Takara, Dalian, China), and the digested DNA was electrophoresed on 0.8% agarose gel. DNA fragments were transferred onto a nylon membrane (Solarbio, Beijing, China) and hybridized with a labelled probe (514 bp) obtained by PCR amplification with primers Yzb-F/R from plasmid pSilent-1.

### 4.8. Data Analysis

Statistical analyses were performed with SPSS 19.0. Statistical differences between treatments were evaluated by Fisher’s least significant difference test at a 5% significance level.

## Figures and Tables

**Table 1:** Effects of enzyme on protoplast yield.

Treatment ^1^	Protoplast Yield (10^7^ Protoplasts/mL)
0.25% L	2.07 ± 0.02b
0.25% D	0.17 ± 0.02d
0.25% S	0.03 ± 0.02d
0.25% S + 0.25% L	2.10 ± 0.28ab
0.25% S + 0.25% D	1.38 ± 0.17c
0.25% L + 0.25% D	2.37 ± 0.27ab
0.25% S + 0.25% D + 0.25% L	2.68 ± 0.24a

^1^ Mycelia lysate: 1 mol/L sorbitol. Data represent the mean ± standard errors (SE) of three independent experiments. Different lowercase letters indicate significant differences (*p* < 0.05).

**Table 2:** Experimental design using Box–Behnken design, experimental value, and predicted value.

Run	X_1_	X_2_	X_3_	Experimental Value (10^7^ Protoplasts/mL) ^a^	Predicted Value (10^7^ Protoplasts/mL)	Residual
1	0	−1	−1	8.05	8.07	−0.02
2	−1	0	−1	5.69	5.77	−0.08
3	1	−1	0	9.14	9.41	−0.27
4	0	0	0	9.33	9.37	−0.04
5	1	0	−1	8.21	7.93	0.28
6	1	0	1	8.69	8.59	0.10
7	−1	−1	0	5.97	5.85	0.12
8	−1	0	1	5.43	5.71	−0.28
9	0	−1	1	8.76	8.57	0.19
10	1	1	0	8.02	8.13	−0.11
11	0	0	0	9.23	9.37	−0.14
12	0	0	0	9.56	9.37	0.19
13	0	1	−1	7.84	8.03	−0.19
14	0	1	1	8.15	8.13	0.02
15	−1	1	0	6.92	6.65	0.27

^a^ Data in the table are the average of three replicates. The other conditions were different concentrations of lysing enzyme to digest 0.035 g mycelia in 90 rpm; the mycelia of *G. tritici* were cultured in OB medium.

**Table 3:** Analysis of variance (ANOVA) of the quadratic model.

Source	Sum of Squares	DF ^a^	Mean Square	*F* Value	*p*-Value Prob > *F*
Model	24.85	9	2.76	29.06	0.0009
X_1_	12.63	1	12.63	132.87	<0.0001
X_2_	0.12	1	0.12	1.29	0.3076
X_3_	0.19	1	0.19	2.02	0.2142
X_1_X_2_	1.07	1	1.07	11.27	0.0202
X_1_X_3_	0.14	1	0.14	1.44	0.2838
X_2_X_3_	0.04	1	0.04	0.42	0.5451
X_1_^2^	8.62	1	8.62	90.72	0.0002
X_2_^2^	0.41	1	0.41	4.31	0.0926
X_3_^2^	2.61	1	2.61	27.45	0.0034
Residual	0.48	5	0.095		
Lack of Fit	0.42	3	0.14	4.86	0.1752
Pure Error	0.057	2	0.029		
Total	25.32	14			

^a^ DF: Degree of Freedom, *R*^2^ = 0.9812, adjusted *R*^2^ = 0.9475, and CV = 3.89%.

**Table 4:** Transformation efficiency of *G. tritici*.

Amount of Transform DNA (µg)	Amount of Transformants	Transformation Efficiency (Transformants/µg DNA)
4	185.00 ± 5.51	46.25 ± 1.38b
6	324.00 ± 3.61	54.00 ± 0.60a
8	391.33 ± 4.33	48.92 ± 0.54b

Data represent the mean ± standard errors (SE) of three independent experiments. Different lowercase letters indicate a significant difference (*p* < 0.05).

**Table 5:** Independent variables, symbols, and levels used in this Box–Behnken design.

Symbols	Independent Variables	−1	0	1
X_1_	Temperature (°C)	28	30	32
X_2_	Enzyme lysis time (h)	2	2.5	3
X_3_	Lysing enzyme concentration (%)	0.5	1	2

## Supplementary Materials

The following are available online at http://www.mdpi.com/1420-3049/23/6/1253/s1, Table S1: The effect of hygromycin B on the mycelial growth of *G. tritici*.
