# Supplementary material for: Optimization of Production Conditions for Protoplasts and Polyethylene Glycol-Mediated Transformation of Gaeumannomyces tritici
Source: Molecules. 2018 May 24;23(6):1253. doi: 10.3390/molecules23061253 (PMC6100196; doi:10.3390/molecules23061253)
Supplement: Supplementary file 1 [file molecules-23-01253-s001.pdf]

Optimization of Production Conditions for Protoplasts and Polyethylene Glycol-Mediated Transformation of *Gaeumannomyces tritici*

Mei Wang [1], Jie Zhang [1], Lanying Wang [1,2], Lirong Han [1], Xing Zhang [1,3,*] and Juntao Feng [1,3,*]

[1] Research and Development Center of Biorational Pesticide, Northwest A&F University, Yangling 712100, China; mmeiwang@126.com (M.W.); deit1984@sina.com (J.Z.); daivemuwly@126.com (L.W.); hlr4119@126.com (L.H.)

[2] Institute of Tropical Agriculture and Forestry, Hainan University, Haikou 570228, China

[3] Engineering and Research Center of Biological Pesticide of Shaanxi Province, Yangling 712100, China

* Correspondence: zhxing1952@gmail.com (X.Z.); fengjt@nwsuaf.edu.cn (J.F.); Tel.: +86-029-87092122

Table S1 The effect of hygromycin B on the mycelial growth of *G. tritici*.

Compounds	Concentrations (μg ml^{-1})	Inhibition(%), mean ± SD	Regression equation (Y =)	EC_{50} (μg ml^{-1}) (95% confidence limit)	*r*
Hygromycin B	10	37.31 ± 2.14	3.39 + 1.22x	21.92 (18.11-25.78)	0.9863
	20	44.78 ± 1.47			
	40	63.06± 1.39			
	60	71.81 ± 2.10			
	80	77.03 ± 1.05			
	100	85.13 ± 1.21			

Data represents the mean value of triplication. The EC_{50} was assessed based on log-transformation analysis.
